# The complete chloroplast genome of a species *Cansjera rheedei* (Opiliaceae)

**DOI:** 10.1080/23802359.2019.1703582

**Published:** 2019-12-18

**Authors:** Guan-Song Yang, Lei Peng, Yue-Hua Wang, Shi-Kang Shen

**Affiliations:** a College of Horticulture and Landscape, Yunnan Agricultural University, Kunming, China;; b School of Life Sciences, Yunnan University, Kunming, China

**Keywords:** *Cansjera rheedei*, chloroplast genome, Opiliaceae

## Abstract

In this study, *Cansjera rheedei* J. F. Gmelin is an important role in the phylogeny and evolution of Opiliaceae plant. The chloroplast genome of *C. rheedei* is 144,306 bp in size, with an average GC content of 37.5%. The complete chloroplast genome has a typical quadripartite structure, including a large single copy (LSC) region (82,773 bp) and a small single copy (SSC) region (9745 bp), which were separated a pair of inverted repeats (IRs, 25,894 bp). This plastome contained 101 different genes, including 67 protein-coding genes (PCGs), 30 tRNA genes and four rRNA genes. The chloroplast genome of *C. rheedei* has completed that will be based on the phylogeny and genomic studies in the family Opiliaceae.

*Cansjera rheedei* is the only species of the genus *Cansjera* in the family Opiliaceae in China. The species is climb lianas or erect shrubs. It is endemic in the forests and thickets in Guangdong, Guangxi, Hainan and Yunnan provinces (Xu et al. 2010). The plant is a conservation of rare plants because of excess utilization and habitat destruction. *Cansjera* plays an important role in the phylogeny and evolution of Opiliaceae. Chploroplast genome of Opiliaceae has not been pubished so far. The chloroplast genome of *C. rheedei* has completed that will accordingly facilitateour understanding of the chloroplast genome feature of Opiliaceae (Yang, Wang et al. 2017). An improved understanding of its genetics would contribute to the formulation of evolutionary direction(Yang, Yang et al. 2017).

Fresh leaves of *C. rheedei* were collected from Jianshui, Yunnan province, China (geospatial coordinates: 23.638418 N, 103.057939 E; altitude: 1123 m), and were used for total genomic DNA. The total genomic DNA was extracted following CTAB method (Doyle and Doyle 1987), then sequenced using the Illumina Hiseq 4000. The Total DNA samples (ZJ 1-1) and the specimens (ZJ 2017-1) were kept at the of College of Horticulture and Landscape herbarium, Yunnan Agricultural University, Kunming, China. The chloroplast genome uses the script Get organelle-reads. The isolated total genomic DNA was fragmented according to the manufacturer’s manual to construct a short insert (500 bp) reads. To directly measure total genomic DNA. Filter out the original reads through the the website (https://github.com/Kinggerm/GetOrganelle). The script calls the spaces for assembly, assembles the filtered readings into contigs, then uses the bandage to connect the contigs, manually removes the extra contigs and connects them to the loop, and finally performs the same reads. It is then remapped to the genome for inspection, proofreading and repair to obtain the final circular chloroplast genome (Bankevich et al. 2012). The Spliced chloroplast genomes were used in Geneious R8 software (Kearse et al. 2012). Sequencing was performed on the Illumina HiSeq X-Ten instrument from Huada company. Mapping PE sequences to assembled plastids using Bowtie2 and Geneious version 9.1.4 software. Manual correction of start and stop codons and intron/exon boundaries were used in Geneious version 9.1.4. (Wyman et al. 2004). All tRNA genes were calibrated by using the tRNAscan-SE online service. The complete plastome was submitted to GenBank (accession number MN_688989). Draw a plastid physical map using Organellar Genome DRAW (Lohse et al. 2013). To determine the phylogenetic location of *C. rheedei* and reconstruct the phylogeny of the Santalales, including *Viscum album* (NC_028012), *Osyris alba* (NC_027960), *Viscum album* (KT_003925), *Osyris alba* (KT_070882.1) and *Champereia manillana* (KY_436366). The plastomes of *Fagopyrum tataricum* (NC_027161) and *Drosera rotundifolia* (KU_168830) was used as out-groups. The maximum-likelihood (ML) phylogenetic tree was reconstructed by using MAFFT (version 7) and RA × ML version 8.1 (Katoh and Standley 2013; Stamatakis 2014), including tree robustness assessment using 1000 replicates of rapid 4 bootstrap with the GTRGAMMA substitution model.

The chloroplast genome of *C. rheedei* was 144,306 bp in size, with an average GC content of 37.5%. The complete chloroplast genome has a typical quadripartite structure, including a large single copy (LSC) region (82,773 bp) and a small single copy (SSC) region (9745 bp), which were separated a pair of inverted repeats (IRs, 25,894 bp). This plastome contained 101 different genes, including 67 protein-coding genes (PCGs), 30 tRNA genes and four rRNA genes.

To determine the phylogenetic location of *C. rheedei*, the maximum likelihood (ML) phylogenetic tree reconstructed based on the whole genomes fully resolved phylogenetic relationships of the three sampled species of Santalales (Figure 1). The chloroplast genome of *C. rheedei* has completed that will be based on the phylogeny and genomic studies in the family Opiliaceae.

**Figure 1.**
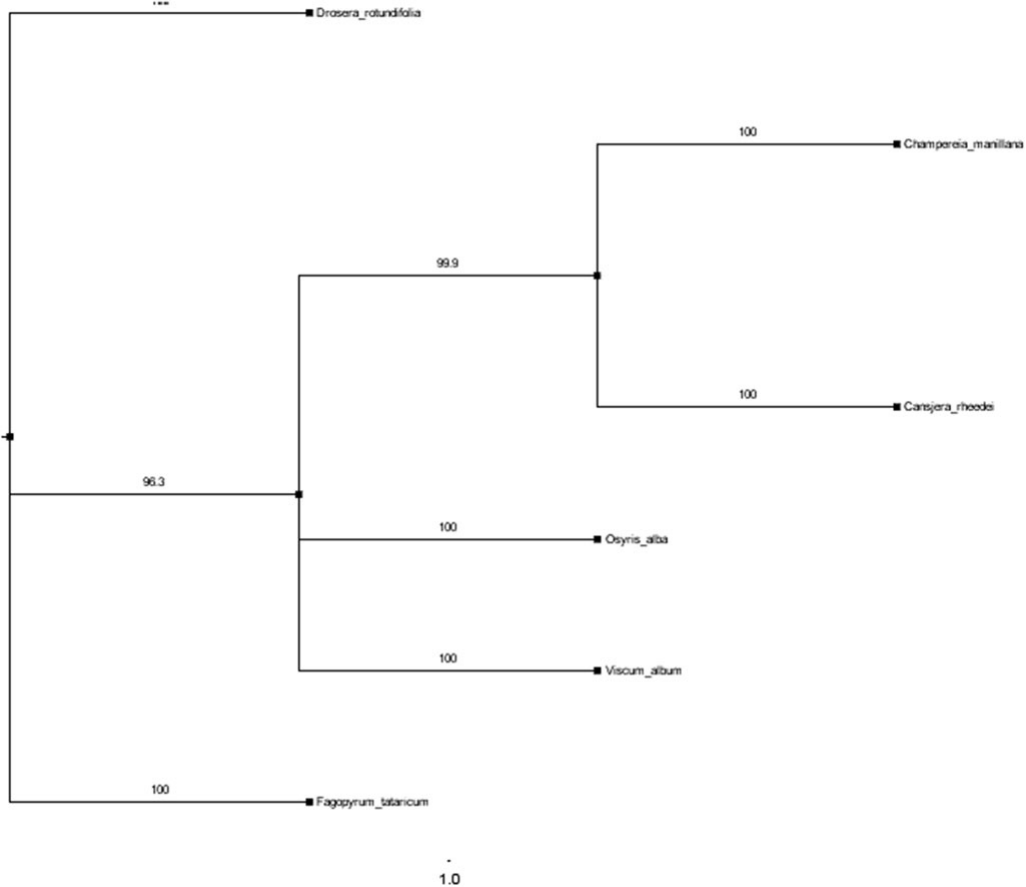
The maximum likelihood (ML) phylogenetic tree based on six complete chloroplast genome sequences. Numbers at the right of nodes are bootstrap support values.
